# Liquid biopsies and those three little words: finding the perfect match for the MTB

**DOI:** 10.1515/medgen-2023-2064

**Published:** 2023-12-05

**Authors:** Adam Wahida, Lars Buschhorn

**Affiliations:** Institute of Metabolism and Cell Death Ingolstädter Landstraße 1 85764 Neuherberg Germany; National Center for Tumor Diseases (NCT) Heidelberg Division of Gynaecological Oncology Im Neuenheimer Feld 440 69120 Heidelberg Germany

**Keywords:** molecular tumor board, MTB, precision oncology, targeted therapy, liquid biopsy, next-generation sequencing, NGS

## Abstract

Monitoring ctDNA by liquid biopsies seems to represent the perfect match for precision oncology and its cornerstone clinical framework: the molecular tumour board (MTB). Detecting and scrutinising the success of targeted therapies or tracking and, for that matter, addressing the therapy with the evolutive nature of a tumour are some of the main advancements one considers to be important for the MTB. One challenge is correlating the estimated allele frequency of each identified genetic alteration determined by analysing the ctDNA sequencing results and matching these with the range of suitable drugs, which may limit the simultaneous treatment of all tumour variations. This limitation arises because a new biopsy would typically be required to evaluate the response to treatment. As a result, evaluating the success of MTB recommendations relies on traditional staging methods, highlighting an existing diagnostic gap. Thus, optimising liquid biopsy technology could enhance the efficacy of MTB treatment recommendations and ensuing tailored therapies. Herein, we discuss the prospect of ctDNA analyses in the molecular tumour board.

## Introduction

As the bridge between modern diagnostics and therapeutics, the molecular tumour board (MTB) represents the hitherto best approximation of applying precision therapy in oncology^[1]^. Herein, an interdisciplinary group typically interprets complex genomic data intending to translate it into actionable clinical recommendations for cancer patients.^[2]^ The perpetual progress of high-throughput sequencing technologies and ensuing targeted therapies has transformed the traditional tumour board model to incorporate molecular profiling data into clinical decision-making, thereby aiming to provide a clinical benefit to its patients^[3,4]^.

Typically, the MTB brings together oncologists, pathologists, geneticists, bioinformaticians, researchers, and other specialists to collaboratively interpret and discuss genomic data from tumour samples^[1]^, which can be an arduous undertaking concerning a lack of precision and evidence regarding the importance and actionability of a particular genetic lesion. Currently, the MTB mainly recommends targeted therapies, immunotherapies, or other appropriate treatments for cases where a particular benefit in rare or refractory tumours is desired. Importantly, the MTB can also identify patients who may be eligible for ongoing clinical trials based on their specific molecular profiles^[5,6]^.

Critical to the interdisciplinary nature of MTBs is that it also fosters a feedback loop, where challenges faced in the clinic can inform research questions and vice versa, thereby transforming the MTB into a “midwife” for translational medicine and the broad application of innovative therapies. Several aspects of this feedback loop are important to elaborate on and consequently reveal a set of challenges the MTB faces in its evolution to become a full-fletched clinical infrastructure with broad application to oncology. Precision oncology’s intricate problems in treating the right patient at the right time with the right drug have been exhaustively discussed elsewhere^[11]^.

One crucial step throughout this journey will be the incorporation of liquid biopsies within the clinical workup of MTB-related therapy recommendations and during therapy^[7]^. By definition, a liquid biopsy is a non-invasive medical test that detects and analyses circulating tumour cells (CTCs), cell-free tumour DNA (ctDNA), and other molecules released by tumours into bodily fluids, primarily blood. This contrasts with traditional biopsies, which require tissue samples from the tumour. Liquid biopsies have gained significant attention in oncology due to their potential to provide valuable information about the genetic makeup and behaviour of cancers without invasive procedures.

In this article, we outline how liquid biopsy profiling can overcome the major hurdles related to providing personalised treatment to cancer patients within the context of an MTB.

## Target engagement: monitoring and guiding therapy response

One of the most obvious issues the MTB faces is if therapeutical targeting of an identified lesion correlates with an expected shrinkage of the respective tumour clone carrying the lesion that is eligible for therapy. One might contemplate several scenarios that might arise when targeting a tumour subclone concerning the clone itself and/or with sister and daughter clones present in a specific patient’s disease. Multiple scenarios are conceivable, such as an actual shrinkage of the targeted clone, as one would in fact, wish. But also, other scenarios are conceivable, such as the non-responsiveness of the clone due to inherent resistance factors like co-occurring mutations or expression patterns hampering therapy success^[1]^.

Of course, the subsection title is posed slightly provocatively since target engagement bears a different meaning per se. Still, one of the main challenges of the MTB is that it is confronted with severe challenges related to measuring granular response. Indeed, while the diagnostic tools used throughout the MTB paint a detailed portrait of an individual tumour’s genomic and or transcriptional aberrancies, the response tools used to monitor therapy success still rely heavily on clinical outcomes and radiological imagery^[8]^. Here, the use of liquid biopsies to precisely assess the allele frequency and, by proxy, the specific burden of a targeted clone seems like the go-to use case of this technology. For instance, changes in the levels of ctDNA in the blood can indicate whether a treatment is effective or if the tumour is developing resistance, for example, as soon as the corresponding allele frequency rises again^[9]^.

Moreover, and beyond the scope of classical radiological surveys, liquid biopsies can be used after initial treatment to detect minimal residual disease (MRD), which refers to the presence of a small number of cancer cells that may be left in the body but are undetectable by conventional methods. Furthermore, molecular testing can be challenging for patients whose tumours are in areas that are difficult to biopsy or for those with health conditions that make tissue collection risky. Liquid biopsy, therefore, offers a potential solution to gather data to direct molecular-based treatment strategies for these individuals.

## Tracking relapse and therapy resistance

As tumours evolve, they might acquire new mutations that make them resistant to previously effective therapies^[10]^. Liquid biopsies can detect these mutations early, which could inform treatment adjustments and re-discussion of the genomic data within the MTB. Logistically attractive and unlike solid biopsies, which are typically done once or occasionally repeated, liquid biopsies can be done more frequently, allowing for more dynamic monitoring of the tumour’s genetic profile and behaviour. This offers the possibility to routinely include genetic testing along with other sero-chemical parameters usually measured from peripheral blood, yielding comprehensive response data critical to conducting subsequent MTB sessions related to a patient.

It’s essential to have supplementary tools for early detection of treatment response and resistance, even before conventional imaging methods are applied during routine clinical follow-up, to guide targeted and chemotherapy treatments effectively. The consistent assessment of ctDNA has been examined as a potential tool for predicting these outcomes. One seminal paper studied 53 patients diagnosed with metastatic colorectal cancer undergoing first-line chemotherapy treatment^[11]^. They monitored the levels of ctDNA in these patients before starting treatment and twice during the first cycle. Notably, they observed significant decreases in ctDNA levels just before the commencement of the second treatment cycle, which correlated with shrinkage of the tumour in CT scans conducted between 8 and 10 weeks into the treatment.

Concurrently, the I-SPY2 TRIAL investigated ctDNA in 61 patients receiving neoadjuvant treatment for breast cancer^[12]^. Their findings revealed that breast cancer patients with detectable ctDNA after neoadjuvant chemotherapy had a greater chance of bearing residual disease. Additionally, individuals who did not achieve a complete pathological response were at a higher risk of recurrence if they tested positive for ctDNA.

Another study explored the ctDNA of colorectal cancer patients who resisted EGFR-blocking therapy initially or after acquiring resistance^[13]^. They identified genetic alterations within the ctDNA of individuals resistant to EGFR inhibitors. Interestingly, they observed a reduction in KRAS mutated alleles in the blood when EGFR-specific antibodies were discontinued, highlighting ongoing genetic changes driven by tumour evolution.

Numerous other studies employed serial ctDNA assessments (occurrence of resistance variants, ctDNA dynamics etc.) to gain insights into the progression of diseases, the development of resistance, and treatment outcomes in various types of cancers, such as colorectal, non-small cell lung, and breast cancer^[14–17]^. These investigations highlighted the emergence of specific mutations or alterations in ctDNA associated with disease progression or resistance to therapeutic interventions.

Nonetheless, liquid biopsies come with their own set of difficulties. The precision and accuracy may differ, and not every tumour releases DNA or cells into the bloodstream at levels that can be noticed. Additionally, there’s the task of differentiating between mutations fueling the tumour’s development and “noise,” referring to either technical anomalies or incidental/passenger mutations. Therefore, it is pivotal to discuss valid cut-offs for clinical interpretation critically. These background mutations are usually derived from mutations due to clonal haematopoiesis (CHIP) and, thus, are of non-tumour origin^[18]^. Therefore, parallel scrutinising the extent of CHIP using Peripheral blood mononuclear cells (PBMCs) might be a viable way to circumvent false-positive results.

Further, mutations that do not correspond to those found during genomic analysis of the available tumor lesion, may be present in ctDNA, due to better representation of the entire tumor. Since ctDNA analysis might add additional information on the tumor molecular profile, complementary liquid biopsy might be of benefit, specifically for patients with distant metastasis. In addition, even non-actionable variants might provide information about ctDNA dynamics per se and could, therefore, be beneficial for monitoring the disease course. Also, mutations that seem pathogenic or affect genes usually associated with cancer can be found in normal tissue or non-malignant disease^[19]^. These detections must also be carefully weighed, posing another layer of complexity upon liquid biopsy profiling. In summary, it will be thus critical to discriminate mutations concerning their tissue of origin, which also bears potential benefits to the application of the MTB since many reported deleterious variants exert differing roles based on the tissue they were identified in.

In conclusion, the regular surveillance of ctDNA offers valuable insights into early responses to treatment and the possible emergence of resistance to targeted therapies and chemotherapy. This data can enhance traditional imaging methods and the use of standard tumor markers^[7]^, particularly within the context of the MTB.

## The holy grail: screening and early detection

Undoubtedly, the penultimate success of the MTB will be achieved when applied early, where tumour evolution has not yet created complexities beyond therapeutic grasp, and genetic screening tools such as liquid biopsies will be required. This also represents the time at which the tumour is still more likely to be curable and, at the same time, before it becomes detectable through imaging or clinical examination or even before it causes symptoms. Nevertheless, many steps must be taken before achieving this goal. These early detection efforts using liquid biopsies are undoubtedly the subject of intense research. Still today, liquid biopsies can be used at best for monitoring therapeutic interventions, surgical or pharmacological, while their use for screening purposes remains at the far end.

Early diagnosis and treatment can significantly enhance outcomes for many cancers. Recognising individuals with early-stage cancers that can be treated with surgery or radiation offers the best possibility for lasting remissions. For instance, the CancerSEEK test employed ctDNA to assess a vast group of patients with identified nonmetastatic cancers. It evaluates the levels of eight proteins and mutations in 1933 distinct genomic positions. Detection accuracy varied between 69 % to 98 % for ovarian, liver, stomach, pancreatic, and oesophageal cancers, and it exhibited a specificity exceeding 99 %. There are currently no screening tests for these cancers, so ctDNA might pave the way for early detection^[20]^.

Many tumours often start with heightened DNA methylation of tumor-suppressing genes. A marker specific to hepatocellular methylation was crafted for cancer diagnosis and monitoring. This set showcased greater accuracy and specificity than AFP for hepatocellular carcinoma detection and matched well with the cancer’s stage, especially for early stages. A seminal study delved into targeted methylation sequencing in ctDNA for various cancers and ascertained that methylation measurements could rightly detect cancer in about 83.8 % of instances with 99.3 % specificity^[21]^. They also correctly determined the cancer variety in about 78.9 % of cases. Another study used a targeted methylation study of ctDNA in over 6600 samples and achieved 99 % specificity and 67 % sensitivity for detecting stage I-III of 12 prevalent cancer types. Pioneering research from Chen and colleagues introduced PanSeer, a blood test for cancer screening that identifies unique cancer-related methylation patterns. Impressively, this test could detect cancer in 95 % of people who showed no symptoms, even up to four years before they were diagnosed^[22]^.

Not only detection of mutant ctDNA or methylation profiles improve early diagnosis. Cristiano and colleagues describe an approach that evaluates fragmentation patterns of cell-free DNA across the genome. They found that patients with cancer had altered profiles and analysed 236 patients with breast, colorectal, lung, ovarian, pancreatic, gastric or bile duct cancer compared to 245 healthy individuals. A machine-learning model showed detections ranges from 57 % to >99 % across the investigated cancer types at 98 % specificity^[23]^. Similar results were published for patients at risk for lung cancer^[24]^.

In conclusion, ctDNA, DNA methylation and ctDNA fragmentomics are all under investigation as potential tools for cancer screening. Untargeted methods especially hold great promise for robust early detection of cancers since they account for the heterogeneous complexity of solid tumours and are not limited in cases lacking hotspot variants. Liquid biopsies might offer a promising avenue to detect early-stage cancers, enabling timely treatments and possibly averting the progression to advanced stages.

## Current limitations of LB in the MTB

Before the widespread adoption of liquid biopsy-based response assessment, it is critical to address several concerns that have not yet been addressed throughout this article. one significant obstacle involves determining how to manage patients with low levels of ctDNA. Another complex subset of patients demanding further evaluation pertains to those with brain tumours or exclusive brain progression. However, several advancements in the detection of ctDNA in the cerebrospinal fluid have been made^[25,26]^. The variability in how ctDNA responds to different treatment methods is another significant limitation. While there is support for the idea that ctDNA reacts predictably regardless of the treatment used, there is still a need for more debate on whether the same ctDNA response benchmarks should be applied across various clinical methods.

## Concluding remarks

The forthcoming adoption of ctDNA to monitor precision oncological approaches represents an exciting development that intertwines with clinical frameworks like the molecular tumour board (MTB). This synergy empowers oncologists to harness the identification of diverse genetic anomalies driving the tumour’s course, allowing them to calibrate and address multiple tumour lineages synchronously or sequentially meticulously. This approach strives to administer treatments aligned with the dynamic nature of the tumour. The precise allele frequency of each discerned genetic variation is ascertained through sequencing outcomes, facilitating an evaluation of the tumour’s clonality sampled through a biopsy.

While the availability of suitable drugs concurrently targeting these different tumour clones is limited, a more pivotal obstacle lies in gauging a drug’s real-time efficacy. This challenge necessitates a fresh biopsy for accurate assessment. Consequently, the effectiveness of MTB recommendations is currently evaluated through conventional staging methods, underscoring the unmet need for refined diagnostic tools. Here, we reviewed the notion that refining liquid biopsy techniques could potentially elevate the precision oncology treatment paradigm^[1]^.

Amid these challenges, the potential of liquid biopsies to reshape cancer care seems promising, enabling early detection, personalised treatment blueprints, and nuanced surveillance of disease and tumour dynamics. Novel applications and scenarios in the landscape of liquid biopsy profiling encompass alternate fluids (cerebrospinal fluid (CSF), ascites, effusions, urine, etc.) and exploring additional analytes such as circulating tumour RNA, cell‐free micro-RNA, and exosomes. Detecting cancer in its incipient, curable phase remains an imperative subject of intense inquiry. The amalgamation of precision oncological methodologies like the MTB with versatile, precise diagnostic prospects offered by liquid biopsies will serve as a launching pad aimed at improved therapies for our afflicted cancer patients. Achieving this conceptual realisation will mandate strides in bioinformatics and sequencing technologies, which are imperative to attaining the envisioned goal.
